# Evaluation of the bioprotectivity of *Lactobacillus* binary/ternary cultures in yogurt

**DOI:** 10.1002/fsn3.1801

**Published:** 2020-07-26

**Authors:** Nasrin Fayyaz, Fakhri Shahidi, Sahar Roshanak

**Affiliations:** ^1^ Department of Food Science and Technology Faculty of Agriculture Ferdowsi University of Mashhad Mashhad Iran

**Keywords:** antifungal activity, Lactic Acid Bacteria, physicochemical properties, yeast, yogurt

## Abstract

The attempts toward addition of biocontrol agents in dairy products have gained popularity. Here, we worked on analysing the antifungal activity of binary and ternary combinations of three Lactic Acid Bacteria (LAB) against five spoilage yeasts in yogurt. The yogurt samples were characterized in terms of pH, acidity, WHC, textural parameters, viscosity, survivability and antifungal activity of LAB and sensorial properties during cold storage. The results showed that the inoculation of LAB in yogurt gave rise in significant reduction of pH throughout cold storage while titrable acidity and WHC decreased (*p* < .05). Inoculation of LAB resulted in significant increase in hardness and adhesiveness while springiness remained constant. On the other hand, apparent viscosity of all samples experienced a profound increase up to the 10th day of storage followed by a reduction trend for the rest of storage period. Analysis of inhibitory activity of LAB showed an efficient barrier against all five yeasts, in which the most activity was recorded for *Lactobacillus reuteri* followed by *Lactobacillus acidophilus*. On the other hand, the most resistance yeast was *Kluyveromyces marxianus* followed by *Rhodotorula mucilaginosa*. Sensorial analysis revealed that addition of LAB in yogurt brought about a profound improvement in textural quality of samples. Inoculation of LAB cultures in yogurt at 5% (v/v) not only could improve the physicochemical and sensorial properties of yogurt, but also could introduce a strategy toward substituting of chemical preservatives with biocontrol agents.

## Graphical abstract

Inoculation of Lactic Acid Bacteria cultures in yogurt at 5% (v/v) not only could improve the physicochemical and sensorial properties of yogurt, but also could introduce a strategy toward substituting of chemical preservatives with biocontrol agents.

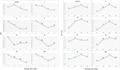

## 1 INTRODUCTION

Dairy industry is one of the most important sections in the food supply chain. The perspective of this industry toward providing the products which can meet the highest physicochemical, nutritional, and microbiological standards has always been affected by the presence of pathogenic and/or spoilage microorganisms. Many attempts have been made in removing or diminishing these microorganisms by using different approaches, including heat treatment (Glosson et al., 2015), high hydrostatic pressure (dos Santos Gouvea et al., 2019), filtration (Sørensen, Jensen, Ottosen, Neve, & Wiking, 2016), and chemical preservatives (Lucera et al., 2014). Considering the adverse effects of the above‐mentioned methods on the nutritional and functional properties of milk compounds, attention has been paid to the use of bioprotective routes by applying the bacteria that have the ability to produce metabolites, called “biopreservative.” The metabolites produced via biopreservative improve both the safety and shelf‐life of the final product. Regarding to the importance of bioprotectivity as a substitute for chemical preservative, several researches have been conducted during last years (Gómez‐Torres, Ávila, Delgado, & Garde, 2016; Leyva Salas et al., 2017). Lactic Acid Bacteria (LAB) are well‐known biopreservative which play their role by producing different compounds such as alcohols, acids, H_2_O_2_, CO_2_ and bacteriocins (Moghanjougi, Bari, Khaledabad, Almasi, & Amiri, 2020), bringing about antibacterial activity and increasing the acidity (Leyva Salas et al., 2017). The inhibitory activity of LAB toward fungal and microbial growth could interpret through three mechanisms including production of organic acids, occurrence of nutrient competition and secretion of antagonistic compounds (Schnürer & Magnusson, 2005). The bioprotectivity of LAB in the dairy industry has been investigated by several researchers for cheese (Angiolillo, Conte, Zambrini, & Del Nobile, 2014; Sedaghat, Eskandari, Moosavi‐Nasab, & Shekarforoush, 2016) and yogurt (Delavenne, Cliquet, Trunet, Barbier, & Le Blay, 2015; Delavenne et al., 2013; Li et al., 2013). Fermentation of food has been used as a method of preservation for centuries, and LAB reduce mold growth and fungal contamination (Mokoena, Chelule, & Gqaleni, 2006).

Using of LAB in dairy products can increase the shelf‐life and overall quality of this products and also can respond to the society's need for chemical‐free, less processed, and safe products on the other hand. The aim of this study was to assess the antifungal activity of binary and ternary combinations of *Lactobacillus helveticus*, *Lactobacillus reuteri* and *Lactobacillus acidiphilus* in yogurt followed by investigating the product in terms of antifungal activity against five common yeasts in yogurt (*Debaryomyces hansenii*, *Rhodotorula mucilaginosa*, *Kluyveromyces marxianus*, *Kluyveromyces lactis*, and *Yarrowia lipolytica*), LAB population, water holding capacity (WHC), viscosity, texture, and sensory properties.

## 2 MATERIALS AND METHODS

### 2.1 Microorganisms and culture conditions

*Lactobacillus reuteri* (IBRC‐M10755), *L. helveticus* (IBRC‐M No 10874), and *Lactobacillus acidophilus* (IBRC‐M No 10815) were supplied from the Iranian Biological Research Center (IBRC) and cultured in the de Man–Rogosa–Sharpe (MRS) broth (Land Bridge Technology Co.) at 37°C for 24–48 hr. For long‐term storage, all strains were maintained in MRS supplemented with 50% (v/v) glycerol at −80°C. *Debaryomyces hansenii* (IBRC‐M No 30329), *R. mucilaginosa* (IBRC‐M No 30357), *K. marxianus* (teleomorph) (IBRC‐M No 30114), *K. lactis* (IBRC‐M No 30241), and *Y. lipolytica* (IBRC‐M No 30168) were cultured in Potato Dextrose Broth (PDB) at 25°C for 48–72 hr.

### 2.2 Antifungal activity assays

In this study, the overlay and the agar well diffusion methods were applied to assess antifungal activity. The overlay method was performed using MRS agar plates on which the LAB were inoculated as two 2‐cm‐long lines and incubated at 30°C for 48 hr in anaerobic jars. The plates were then overlaid with 9 ml of PDA and 1 ml of yeast. The plates were then incubated aerobically at 30°C for 48 hr. Then, the clear zones of inhibition around the bacterial streaks were examined and the area of the zones were scored (Magnusson & Schnürer, 2001).

For the microdilution method, active isolates were inoculated in a 100 ml conical flask containing MRS broth and incubated at 30 ± 2°C for 48 hr. After that, the suspension was centrifuged (12,500 *g*, 10 min, 4°C) and the supernatant was collected and filtered through 0.22 μm membrane filters. 190 μl of the isolate supernatants and 10 μl of the conidial suspensions were dispensed in 96 wells. All the experimental plates were incubated at 30 ± 2°C for 72 hr. Fungal growth was measured at 600 nm using a microplate reader. Here, the growth of the fungi in the control was considered 100% growth. Based on the percentage of the fungal growth, inhibition was calculated. The sample free of the fermentative metabolites was considered as control (Ilavenil et al., 2015).

### 2.3 Yogurt preparation

The yogurt samples were prepared using milk with 1.5% fat, 8.1% no fat milk solids, and a pH value of 6.63. After heat treatment (85°C, 30 min), the milk was rapidly cooled down to 45°C before adding 0.05% w/v of the thermophilic yoghurt culture (*Streptococcus thermophilus*, *Lactobacillus delbrueckii* subsp. *lactis* and *L. delbrueckii* subsp. *bulgaricus*, CHR Hansen). The adjunct three LAB bacteria ~ 10^8^ CFU/ml (*L. acidophilus*, *L. helviticus*, *L. returi*) was added to the milk at a concentration of 5% (v/v) (Table 1). Fermentation was conducted at 42°C for 5 hr, and the samples were stored at 4°C for 30 days.

**TABLE 1:** Binary and ternary combinations of Lactic Acid Bacteria (LAB) based on the milk volume (L) and 0.05% Hansen starter

Sample code	LAB (% v/v)		
	*Lactobacillus helveticus*	*Lactobacillus reuteri*	*Lactobacillus acidophilus*
1	5%	0	0
2	0	5%	0
3	0	0	5%
12	2.5%	2.5%	0
13	2.5%	0	2.5%
23	0	2.5%	2.5%
123	1.66%	1.66%	1.66%

### 2.4 Determination of pH and acidity

The pH values of the samples were measured using a digital pH meter (HANNA, pH 211) at 27°C. For determination of the titratable acidity (TA), a certain amount of each sample was mixed with 10 ml of hot distilled water and titrated with NaOH 0.1 N in the presence of 0.5% phenolphthalein indicator (Li *et al*., 2013).

### 2.5 Water holding capacity

The weight of an empty centrifuge tube was recorded, and then, 15 ml of each sample was poured into it, after that the entire weight was recorded. The sample was centrifuged (Anting, LXJ‐IIB) at 4,000 *g* for 20 min, and the supernatant was drained. The centrifuge tube was inverted for 10 min, and its weight was measured. WHC (%) was calculated using equation (1):

$$\text{WHC}=W/W_{0}\times 100,$$

where *W* and *W*_0_ are the weight of the precipitate and the weight of the yogurt, respectively (Amal, Eman, & Nahla, 2016).

### 2.6 Apparent viscosity

A rotational Bohlin viscometer (Visco 88, Bohlin Instruments) equipped with a Julabo heating circulator F12‐MC (Julabo Labortechnik) and a measuring cylindrical spindle (C30) was used to determine the apparent viscosity. Before the apparent viscosity measurements, the samples were treated under a shear rate of 100/s for 30 min at 25°C so as to minimize the time dependency effect. Eventually, the yogurt samples viscosity was assessed at shear rates ranging from 14.4 to 296/s at 25°C and the apparent viscosity was reported at a shear rate of 51.5/s (Morris, 1994).

### 2.7 Texture profile analysis

The yogurt samples were stored at 20°C for 10 min followed by being analyzed using a Brookfield texture analyzer (CT V1.5 Texture Analyzer; Brookfield) equipped with a cylindrical probe, 20 mm in diameter. The TPA measurements were conducted at 2 mm/s penetration speed up to a depth of 20 mm. The textural parameters, including hardness (g), adhesiveness (mj), cohesiveness, springiness, gumminess (g), and chewiness (mj), were studied for each sample.

### 2.8 Enumeration of LAB in yogurt

One gram of each yogurt sample was diluted with 99 ml of Ringer solution. Subsequent 10‐fold serial dilutions were made with ringer, and 0.1 ml of the diluted samples was spread on MRS agar. After anaerobic incubation at 37°C for 48–72 hr, and enumeration, the number of LAB was calculated as CFU/g. This method was repeated on days 10, 20, and 30 by the initial cell count on day 0.

### 2.9 Sensory analysis

A trained panel of 15 assessors evaluated the samples in terms of texture, color, and taste. A 100‐point scale was used to evaluate the sensory properties of the yogurt samples.

## 3 RESULTS AND DISCUSSION

### 3.1 pH

**FIGURE 1:**
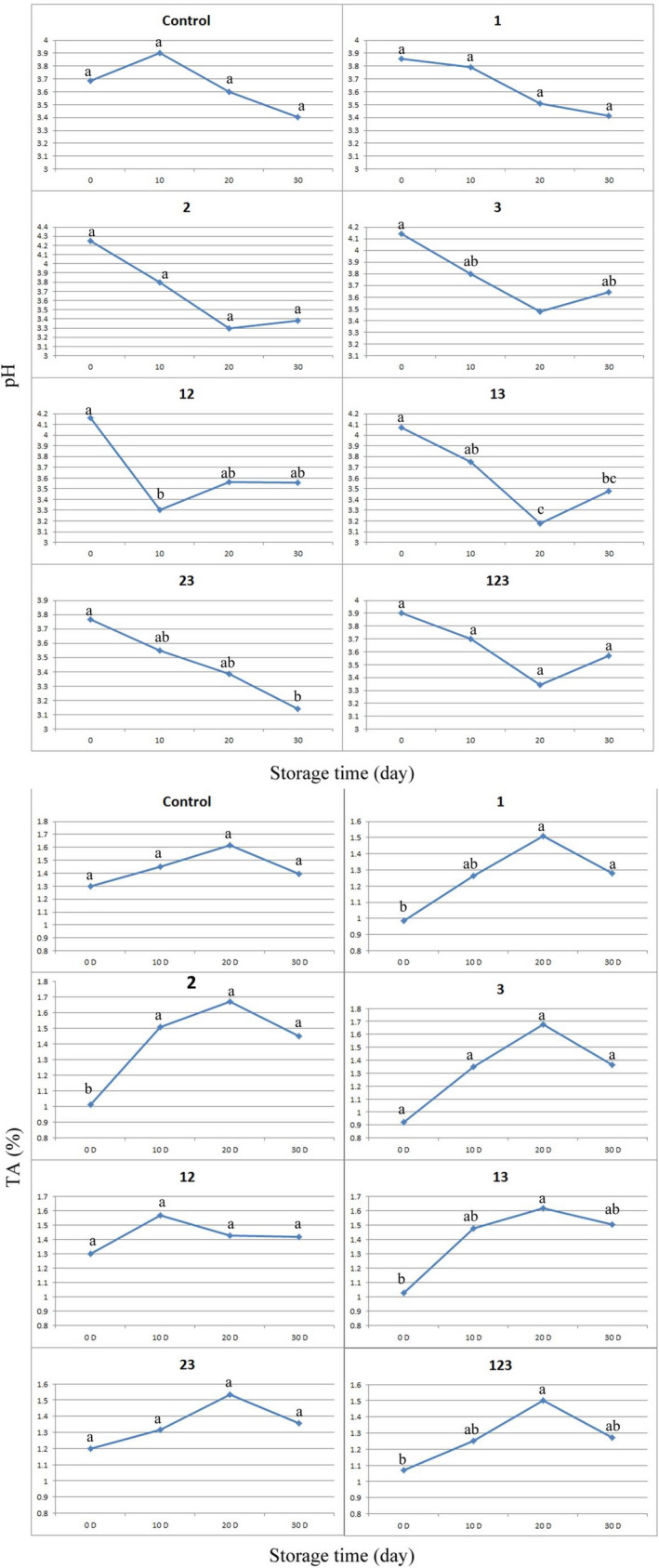
pH and titratable acidity (TA) variation in yogurt samples prepared with different combination of Lactic Acid Bacteria (LAB) during storage

Figure 1 depicts the decreasing trend of yogurt pH in parallel to increase the TA during storage in which the minimum and maximum changes were, respectively, recorded for control and sample 2 and revealed the higher fermentation degree in LAB included samples as well as their survival during cold storage. These results showed that *L. reuteri* had the highest postacidification capability. Li et al. (2013) reported the same results for yogurt prepared via *Lactobacillus casei* as a bioprotective culture. Contrary to these results, Leyva Salas et al. (2017) reported that inclusion of antifungal culture had no effect on the pH of cream and cheese during 1‐month cold storage period. The reason behind this observation could related to difference in the matrix of products, that have a profound effect on growing of LAB (Leyva Salas et al., 2017). An increasing trend in pH was observed at samples of 2, 3, 13, and 123 that could interpreted through results of Akalın, Unal, Dinkci, and Hayaloglu (2012). According to those authors, the ability of *S. thermophilus* to produce some basic metabolites during the later stage of storage could be a possible reason for the increase in pH observed during storage.

### 3.2 Water holding capacity

The gel network of yogurt is characterized by its dynamic nature affecting by proteins and calcium salt, which chemical and process variables may have sensible influence on it (Ziarno & Zaręba, 2019). The results of variations in WHC of yogurt samples during storage were shown in Table 2. For all LAB added yogurt, WHC was increased significantly with time (*p* < .05), while for control sample, the change was nonsignificant (*p* > .05). The interactions occur during storage periods in terms of acid production due to the LAB growth bring about the casein particles to hold water molecules more efficiently, resulted in syneresis decrementation (Öztürk & Öner, 1999). The proteins WHC incrementing due to decrease in pH in parallel to LAB growth give rise to increase yogurt curd stability (Öztürk & Öner, 1999).

**TABLE 2:** Water holding capacity yogurt samples prepared with different combination of Lactic Acid Bacteria during storage

Sample code	The water holding capacity (%)			
	0 day	10 days	20 days	30 days
0	21.00 ± 1.41^aA^	22.14 ± 0.06^aA^	19.88 ± 0.17^bcA^	21.85 ± 0.19^cdA^
1	16.91 ± 0.41^bB^	20.99 ± 0.15^bA^	17.76 ± 0.34^dB^	20.82 ± 1.18^dA^
2	19.54 ± 0.79^abB^	20.93 ± 0.38^bB^	20.52 ± 0.69^bB^	25.62 ± 0.54^bdA^
3	17.98 ± 0.86^abA^	19.81 ± 0.46^cA^	18.92 ± 0.15^cdA^	20.47 ± 0.82^dA^
12	20.44 ± 0.78^aC^	21.03 ± 0.38^bC^	24.94 ± 0.12^aB^	35.29 ± 0.43^aA^
13	18.75 ± 0.35^abC^	21.96 ± 0.06^abB^	18.90 ± 0.17^cdC^	23.83 ± 0.28^bcA^
23	18.28 ± 1.10^abB^	21.04 ± 0.21^bA^	20.62 ± 0.57^bB^	21.78 ± 0.31^cdA^
123	19.63 ± 0.52^abB^	20.99 ± 0.05^bA^	19.21 ± 0.27^bcdB^	20.79 ± 0.26^dA^

Note

a, b, c, d Significant differences in each row among the means (*p* < .05).

A, B, C Significant differences among the means in each column (*p* < .05).

### 3.3 Survivability of LAB in yogurt sample

The survivability of LAB as a function of storage time is shown in Table 3, in which the viable counts of LAB in the yogurt samples of control, 2, 3, and 23 were changed insignificantly (*p* > .05), while the viable counts in the yogurt samples 1, 13, and 123 were significantly decreased (*p* < .05). Ziarno and Zaręba (2019) reported similar trend toward significant reduction of *Lactobacillus* population during cold storage of yogurt samples. It is reported that the starter culture may have negative effect on the survival of LAB. A work on the effect of *S. thermophilus* or *L. bulgaricus* on the survival of LAB was conducted by Ng, Yeung, and Tong (2011) who reported the negative impact of *S. thermophilus* and *L. bulgaricus* on the growth of LAB. This trend could attribute to the metabolites producing by starter culture (Sadiq et al., 2019).

**TABLE 3:** The survivability of Lactic Acid Bacteria (LAB) as a function of storage time

Sample code	Log of LAB in CFU/g			
	0 day	10 days	20 days	30 days
Control	5.46 ± 0.08^cA^	5.97 ± 0.09^bcA^	5.07 ± 0.52^dA^	5.61 ± 0.31^bcA^
1	6.34 ± 0.05^abcA^	5.86 ± 0.14^bcB^	5.85 ± 0.09^bcB^	5.83 ± 0.10^bcB^
2	6.84 ± 0.84^abA^	5.81 ± 0.04^bcA^	5.36 ± 0.05^cdA^	5.39 ± 0.03^bcA^
3	5.86 ± 0.10^bcA^	6.13 ± 0.06^bA^	6.09 ± 0.07^abcA^	5.68 ± 0.44^cA^
12	6.69 ± 0.12^abB^	6.16 ± 0.15^bC^	6.87 ± 0.12^aAB^	7.29 ± 0.04^aA^
13	6.49 ± 0.07^abcA^	4.97 ± 0.16^dB^	5.65 ± 0.06^bcdAB^	5.65 ± 0.38^bcB^
23	6.25 ± 0.03^abcB^	6.98 ± 0.07^aA^	6.20 ± 0.03^abB^	5.95 ± 0.21^bcB^
123	7.22 ± 0.06^aA^	5.66 ± 0.01^cC^	6.04 ± 0.23^bcBC^	6.34 ± 0.20^abB^

Note

a, b, c, d Significant differences in each row among the means (*p* < .05).

A, B Significant differences among the means in each column (*p* < .05).

The viable counts of yogurt sample 12 were significantly increased during the storage period. In consequence with this results, Leyva Salas et al. (2017) illustrated that the population of biopreservative culture in both cream and cheese followed a stable or in some case increasing trend during cold storage period. The viability of LAB in yogurt is a function of different variables including the possible interactions with starter culture, the amount of produced metabolites such as acid, H_2_O_2_ and dissolved O_2_, strain variation and storage condition (Leyva Salas et al., 2017). The highest viable counts of LAB in sample 12 at the end of storage period, on the one hand, and the increasing trend of *L. helveticus* and *L. reuteri* growth throughout the 30 days storage, on the other hand, could interpret through the synergistic effect of starter culture and added LAB (Sah, Vasiljevic, McKechnie, & Donkor, 2015; Shori, 2015) as well as between the *L. helveticus* and *L. reuteri*. Similarly, (Bian et al. (2016)) reported that inoculation of *L. helveticus* in different levels as antifungal culture in fermented soybean milk, bring about improving the viability of *L. bulgaricus* than control sample during 21 days cold storage.

### 3.4 Antifungal activity

The results of assessment the antifungal activity of LAB by two approaches of overlay and microdilution are shown in Tables 4 and 5. The antifungal activity of *L. reuteri* in both methods was higher than other LAB followed by *L. acidophilus*. The higher antifungal activity of *L. reuteri* could attribute to its ability toward producing dedicate active compound, named reuterin (Vimont, Fernandez, Ahmed, Fortin, & Fliss, 2019). Based on the overlay method, two yeasts, *R. mucilaginosa* and *K. marxianus,* had higher spoilage potential than others so no inhibitory was observed in the presence of *L. helveticus* for both of them. In agreement with our findings, Magnusson and Schnürer (2001) showed that *Lactobacillus coryniformis* could induce a weak suppression against *K. marxianus* and *D. hansenii*. It is reported that the co‐culture of *L. helveticus* and *K. marxianus* was applied in the process of producing some dairy and nondairy products such as fermentation of cheese whey and sourdough, due to their synergistic effect on each other (Banu & Aprodu, 2012; Plessas, Bosnea, et al., 2008; Plessas, Fisher, et al., 2008). Accordingly, it is predictable that no inhibitory zone will be observed in this case. Similarly, the growth of *K. marxianus* was not inhibited in the presence of *L. acidophilus*. The reason behind this observation could interpret through the work of Ahtesh, Apostolopoulos, Stojanovska, Shah, and Mishra (2018) who reported the synergistic effect between *L. acidophilus* and *K. marxianus* in co‐culture medium, in which the growth of yeast was enhanced due to the presence of *L. acidophilus*. On the other hand, minimal deterrence was recorded after *L. reuteri* for *K. marxianus* and *R. mucilaginosa*, and *L. acidophilus* for *R. mucilaginosa*. It can be said that *D. hansenii* were completely suppressed by *L. reuteri* and *L. acidophilus* and *Y. lipolytica* by *L. acidophilus*. On the other hand*, K. lactis* completely lose its growth ability in the presence of *L. reuteri*.

**TABLE 4:** Antifungal activity of Lactic Acid Bacteria (LAB) by overlay method

yeast	LAB bacteria		
	*Lactobacillus reuteri*	*Lactobacillus helveticus*	*Lactobacillus acidophilus*
*Debaryomyces hansenii*	+++	+++	++
*Rhodotorula mucilaginosa*	+	−	+
*Kluyveromyces marxianus*	+	−	−
*Kluyveromyces Lactis*	+++	++	++
*Yarrowia lipolytica*	++	++	+++

Note

−, no suppression; +, no fungal growth on 0.1–3% of the plate area per bacterial streak; ++, no fungal growth on 3–8% of plate area per bacterial streak; or +++, no fungal growth on >8% of plate area per bacterial streak.

**TABLE 5:** Antifungal activity of Lactic Acid Bacteria bacteria by microdilution Method

Tested fungi	*Lactobacillus reuteri*	*Lactobacillus helveticus*	*Lactobacillus acidophilus*
*Debaryomyces hansenii*	74.71 ± 1.60^bA^	35.80 ± 1.20^cB^	73.95 ± 1.90^bA^
*Rhodotorula mucilaginosa*	73.27 ± 0.50^bA^	72.51 ± 0.90^bA^	74.17 ± 2.65^bA^
*Kluyveromyces marxianus*	86.67 ± 1.70^aA^	0.00^dB^	84.91 ± 1.60^aA^
*Kluyveromyces lactis*	90.74 ± 2.50^aA^	0.00^dB^	89.50 ± 1.80^aA^
*Yarrowia lipolytica*	56.76 ± 0.80^cB^	82.35 ± 0.80^aA^	55.85 ± 1.70^cB^

Note

The percentage of fungal growth inhibition was calculated from fungal growth in control. The results were expressed as mean ± *SD* of three replicates.

a, b, c Significant differences in each row among the means (*p* < .05).

A, B Significant differences among the means in each column (*p* < .05).

Table 5 represents the antifungal activity LAB bacteria via microdilution method, in which the most efficient LAB against yeasts is *L. reuteri* followed by *L. acidophilus*, by more than 50% inhibition for all assessed yeasts*. Lactobacillus helveticus* could not inhibit *K. marxianus* and *K. lactis* and their inhibitory against *D. hansenii* was narrowed (~35%), while the most deterrence efficiency of *L. reuteri* and *L. acidophilus* were recorded after *K. marxianus* and *K. lactis*. No significance difference was observed between the antifungal activity of *L. reuteri* followed by *L. acidophilus* for all five yeasts. On the other hand, the inhibitory of *L. helveticus* against *Y. lipolytica* was significantly more than other two strains. These observations support the idea of present work toward using binary and ternary combinations of LAB in order to achieve the highest possible inhibitory effect against fungal growth in yogurt. In agreement with our results, several researchers reported the antifungal activity of LAB in yogurt including *Lactobacillus amylovorus* (Ryan et al., 2011), *Lactobacillus harbisensis* and *Lactobacillus ramnosus* (Delavenne et al., 2015), and *Lactobacillus casei* (Li et al., 2013). Bian et al. (2016) illustrated that *L. helveticus* had the ability to completely inhibit the growth of *Penicillium* sp. in fermented soybean milk during the 28 days of cold storage. In appropriate conditions, LAB are able to produce different antifungal metabolites such as organic acids, phenolic compounds, fatty acids, H_2_O_2_, reuterin, and proteinaceous compounds (Dalié, Deschamps, & Richard‐Forget, 2010). In dairy products, hydrogen peroxide interacts with thiocyanate, resulted in forming intermediatory molecules having the ability to disrupt the growth of unpleasant organisms (Schnürer & Magnusson, 2005). The ability of organic acids toward entering microorganism cells gives rise to reduce the cytoplasmic pH followed by metabolism disruption. On the other hand, lacking the ability of catalase production in LAB bring about the H_2_O_2_ to accumulate in their growth medium give rise to fungal inhibitory activity (Zotta et al., 2018).

The differences observing between the results of LAB antifungal activity by two approaches (Tables 4 and 5) could explain relying on the work conducting by Magnusson, Ström, Roos, Sjögren, and Schnürer (2003) who reported that the extent of antifungal compounds production by LAB was profoundly depended on their growth environment. Similarly, Leyva Salas et al. (2017) illustrated that the medium at which antifungal activity of LAB was assessed had a decision influence toward producing active metabolites. In this regard, Delavenne et al. (2013) and Le Lay et al. (2016) showed that some of LAB just were metabolically active in vitro and were ineffective in product.

### 3.5 Texture profile analysis

Table 6 represents the textural properties of yogurt samples prepared with different type and ratios of LAB. The textural features of yogurt are profoundly affected by process parameters and fermentation variables such as acid production (Ziarno & Zaręba, 2019). Addition of LAB resulted in significant increase in hardness and adhesiveness. The increase in hardness of yogurt during storage can related to the pH reduction caused by LAB activity give rise to change of casein electric charge (Harwalkar & Kalab, 1986). During the storage period, as a result of producing acids followed by pH reduction, the surface charge of casein increase gives rise to yogurt gel to be more rigid. Several researchers reported similar results toward increasing the yogurt hardness by time (Sah, Vasiljevic, McKechnie, & Donkor, 2016). Mani‐López, Palou, and López‐Malo (2014) reported that the firmness of yogurts containing *L. delbrueckii* ssp. *bulgaricus* and *L. reuteri* or *L. acidophilus* increased during 35 days of storage. Penna, Gurram, and Barbosa‐Cánovas (2006) illustrated that starter culture and inoculation rate were determined the fermentation pathways, role playing in textural characteristics of final product. The springiness of yogurt samples varied from 0.75 to 0.95, in which the springiness of control yogurt was decreased significantly while its variation was narrow and insignificant for LAB‐added yogurt, maybe due to the postacidification by LAB during storage. The springiness, defined as the ability of yogurt gel network to recover after the first deformation, is related to the protein–protein bonding, that itself is affected by the rate in which the colloidal phosphates released from casein micelles. These reactions is governed by the acid production rate by starter culture (Sah et al., 2016). The same pattern was observed in terms of gumminess and chewiness. It is reported that starter culture had a meaningful impact on gumminess and chewiness of yogurt (Penna et al., 2006). Adhesiveness is strongly linked to firmness (Hilali et al., 2011). Mani‐López et al. (2014) reported that yogurts with *L. delbrueckii* ssp. *bulgaricus* and *L. reuteri* or *L. casei* increased adhesiveness during storage, and adhesiveness was significantly different between products during storage, at which larger firmness values were generally associated with low adhesion values.

**TABLE 6:** Textural properties of yogurt samples during cold storage

Test	Storage day	Sample							
		Control	1	2	3	12	13	23	123
Hardness (g)	0	23.00 ± 1b	24.00 ± 0b	22.00 ± 1b	24.00 ± 1b	21.00 ± 2b	24.00 ± 0ab	23.00 ± 1b	23.00 ± 1b
	10	25.00 ± a	26.00 ± 1a	24.00 ± 0ab	24.00 ± 1b	24.00 ± 0ab	24.00 ± 1ab	25.00 ± 1ab	25.00 ± 1ab
	20	22.00 ± 0b	24.00 ± 1b	24.00 ± 1ab	26.00 ± 2ab	27.00 ± 1a	25.00 ± 0ab	25.00 ± 1ab	24.00 ± 1ab
	30	20.00 ± 1c	27.00 ± 0a	25.00 ± 1a	28.00 ± 2a	25.00 ± 1ab	27.00 ± 1a	27.00 ± 0a	26.00 ± 1a
Adhesiveness (mj)	0	0.30 ± 0.03b	0.20 ± 0.01b	0.20 ± 0.02bc	0.20 ± 0.01b	0.82 ± 0.03a	0.10 ± 0.02b	0.20 ± 0.01c	0.10 ± 0.02c
	10	0.10 ± 0.01c	0.40 ± 0.01a	0.40 ± 0.03b	0.20 ± 0.01b	0.20 ± 0.02b	0.30 ± 0.03bab	0.30 ± 0.01b	0.30 ± 0.01b
	20	0.90 ± 0.03a	0.20 ± 0.0b	0.50 ± 0.02a	0.30 ± 0.01b	0.70 ± 0.03ab	0.30 ± 0.04ab	0.60 ± 0.02a	0.50 ± 0.02a
	30	0.50 ± 0.03b	0.30 ± 0.03ab	0.50 ± 0.05a	0.80 ± 0.02a	0.70 ± 0.04ab	0.40 ± 0.02a	0.20 ± 0.04c	0.40 ± 0.01ab
Springiness	0	0.91 ± 0.03a	0.89 ± 0.03a	0.95 ± 0.04a	0.91 ± 0.01a	0.91 ± 0.05a	0.92 ± 0.02a	0.90 ± 0.01a	0.81 ± 0.04a
	10	0.93 ± 0.04a	0.90 ± 0.01a	0.90 ± 0.05b	0.90 ± 0.02a	0.90 ± 0.03a	0.94 ± 0.02a	0.91 ± 0.02a	0.83 ± 0.05a
	20	0.83 ± 0.02b	0.89 ± 0.03a	0.95 ± 0.04a	0.90 ± 0.04a	0.86 ± 0.06b	0.92 ± 0.04a	0.84 ± 0.01b	0.83 ± 0.03a
	30	0.84 ± 0.01b	0.90 ± 0.05a	0.94 ± 0.05a	0.91 ± 0.01a	0.91 ± 0.03a	0.92 ± 0.05a	0.91 ± 0.01a	0.81 ± 0.01a
Gumminess (g)	0	20.00 ± 2a	20.00 ± 2a	22.00 ± 3a	21.00 ± 1a	21.00 ± 2a	22.00 ± 1a	19.00 ± 2a	20.00 ± 2a
	10	21.00 ± 1a	19.00 ± 3a	18.00 ± 1b	21.00 ± 2a	22.00 ± 1a	21.00 ± 2a	21.00 ± 1a	20.00 ± 3a
	20	19.00 ± 2a	20.00 ± 2a	23.00 ± 2a	21.00 ± 1a	15.00 ± 3b	20.00 ± 2a	16.00 ± 1b	19.00 ± 1a
	30	13.00 ± 3c	21.00 ± 1a	23.00 ± 1a	20.00 ± 3a	22.00 ± 3a	22.00 ± 3a	20.00 ± 2a	19.00 ± 3a
Chewiness (mj)	0	3.80 ± 0.1a	3.50 ± 0.2a	3.50 ± 0.2a	3.60 ± 0.2a	3.90 ± 0.3a	3.80 ± 0.2a	3.50 ± 0.1a	4.20 ± 0.2a
	10	3.90 ± 0.2a	3.00 ± 0.1b	3.10 ± 0.5b	3.70 ± 0.2a	3.80 ± 0.1a	3.50 ± 0.5a	3.70 ± 0.4a	3.20 ± 0.5b
	20	3.10 ± 0.1b	3.50 ± 0.5a	3.40 ± 0.2a	3.70 ± 0.4a	2.50 ± 0.1b	3.50 ± 0.3a	2.70 ± 0.4b	4.10 ± 0.1a
	30	2.20 ± 0.4c	3.80 ± 0.4a	3.60 ± 0.1a	3.60 ± 0.1a	4.00 ± 0.4a	3.60 ± 0.1a	3.70 ± 0.6a	4.00 ± 0.1a

### 3.6 Viscosity

The apparent viscosity of yogurt samples is shown in Table 7. A similar trend was observed in all samples, in which a significant increase was recorded in day 10 followed by reduction up to the end of storage. Several researchers attributed these changes to the acidity of medium, in which a firmer network was appeared as a result of acid coagulation at low pH (Beal, Skokanova, Latrille, Martin, & Corrieu, 1999; Garcia‐Garibay & Marshall, 1991). It is reported that the factors affecting fermentation pathway and duration could influence the viscosity of final product (Penna et al., 2006). In this study, inoculation of LAB at different ratios resulted in producing different acids with different rates and concentrations, bringing about the apparent viscosity to be a culture dependent feature. In this regard, Beal et al. (1999) reported that the viscosity of yogurt is a function of strain association, temperature, and final pH. In contrary, Li et al. (2013) represented results toward the effectless of inclusion *L. casei* on the apparent viscosity of yogurt samples during the refrigerated storage period. This opposed results can interpret relying on the difference in type and concentration as well as the interactions occurred between binary and ternary cultures.

**TABLE 7:** Apparent viscosity of yogurt sample during cold storage

Sample Storage day	Apparent viscosity (Pas)			
	0	10	20	30
Control	0.067 ± 0.003^bD^	0.176 ± 0.005^dA^	0.157 ± 0.008^bB^	0.105 ± 0.003^aC^
1	0.079 ± 0.004^aD^	0.159 ± 0.003^eA^	0.138 ± 0.005^cB^	0.098 ± 0.006^abcC^
2	0.055 ± 0.003^cdD^	0.250 ± 0.006^aA^	0.093 ± 0.002^eC^	0.104 ± 0.002^abB^
3	0.062 ± 0.001^bcC^	0.097 ± 0.002^fA^	0.086 ± 0.005^eB^	0.084 ± 0.002^dB^
12	0.011 ± 0.004^eC^	0.230 ± 0.007^bA^	0.075 ± 0.002^fB^	0.012 ± 0.001^eC^
13	0.068 ± 0.002^bC^	0.084 ± 0.001f^gB^	0.122 ± 0.004^dA^	0.092 ± 0.004^bcdB^
23	0.063 ± 0.003^bcB^	0.072 ± 0.006^gAB^	0.076 ± 0.002^fAB^	0.089 ± 0.06^cdA^
123	0.051 ± 0.001^dD^	0.216 ± 0.009^cA^	0.187 ± 0.003^aB^	0.083 ± 0.001^dC^

### 3.7 Sensory analysis

**FIGURE 2:**
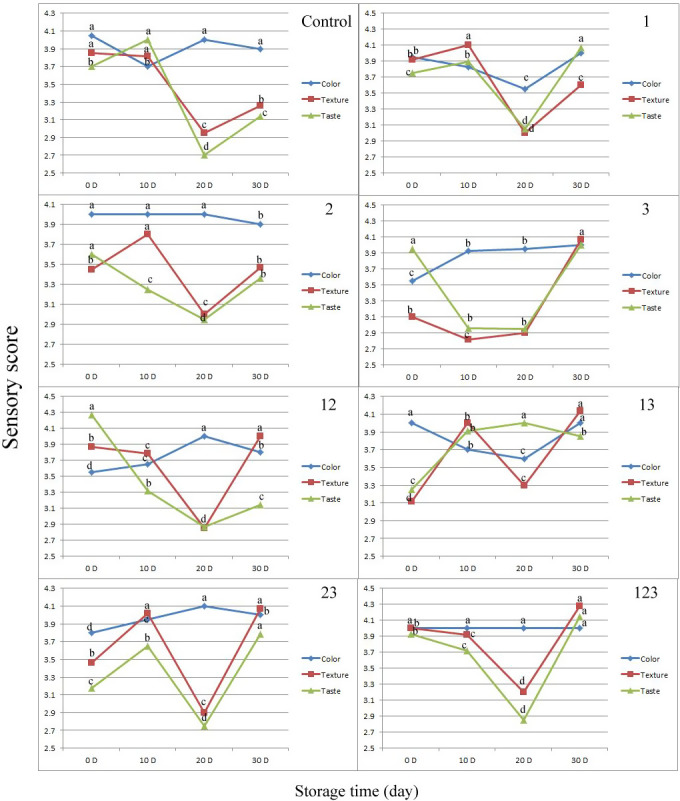
Sensory analysis of yogurt samples prepared with different combination of Lactic Acid Bacteria (LAB) during storage

The impact of LAB cultures on the sensorial characteristics of the yogurt samples was shown in Figure 2. At a glance, the sensorial quality of all LAB included yogurt samples was significantly improved during 30 days of cold storage. The most changes in sensorial scores were come after taste characteristic (Figure 2a). The taste score of LAB‐included yogurt samples was significantly increased during 30 days of storage. These observations could interpret relying on the ability of LAB toward producing several taste related compounds. LAB conduct the biochemical processes including glycolysis, proteolysis, and lipolysis resulting in formation of aldehydes, ketones, acids, alcohols, esters, aromatic hydrocarbons, heterocyclic, furans, and sulfur compounds (Chen et al., 2017). It is reported that inclusion of *L. helveticus* in fermented soybean milk had no significant influence on the sensory quality during 21 days cold storage (Bian et al., 2016). Similarly a report launched by Li et al. (2013) in which the sensory properties of yogurt supplemented via *Lactobacillus casei* had no meaningful difference with blank sample. These differences could attribute to the change in the type and also concentration of LAB included in yogurt in present study in compare to them. In the case of texture, considering the panelists scores and TPA results, it can be concluded that the panelists were preferred yogurt samples having higher hardness, moderate adhesiveness and cohesiveness and lowest gumminess and chewiness. Similarly, Mani‐López et al. (2014) and Hekmat and Reid (2006) reported that panelists did not recognize texture or flavor differences among probiotic and nonprobiotic samples; therefore, probiotic yogurts can be modified using different culture mixtures without sensory complaints.

## 4 CONCLUSION

The bioprotectivity of three LAB, namely *L. reuteri*,* L. helveticus*, and* L. acidophilus*, in the inoculation level of 5% (v/v) in forms of binary and ternary combinations in yogurt was assessed against five spoilage yeasts, namely *D. hansenii, R. mucilaginosa, K. marxianus, K. lactis,* and *Y. lipolytica*. The further LAB added yogurt analysis showed several significant changes including pH reduction, WHC incremention, improvement sensory quality and textural properties, maybe due to the acid accumulation as a result of LAB growth throughout of storage. The investigated LAB had sensible inhibitory against all five yeasts. Our study provided information toward using binary and ternary cultures of three LAB as an efficient route in terms of introducing a biocontrol system in dairy products for simultaneous growth inhibition of a variety of yeasts.

## CONFLICT OF INTEREST

None.

## ETHICAL APPROVAL

This article does not contain any studies with human or animal subjects.
